# *Tosanoidesaphrodite*, a new species from mesophotic coral ecosystems of St. Paul’s Rocks, Mid Atlantic Ridge (Perciformes, Serranidae, Anthiadinae)

**DOI:** 10.3897/zookeys.786.27382

**Published:** 2018-09-25

**Authors:** Hudson T. Pinheiro, Claudia Rocha, Luiz A. Rocha

**Affiliations:** 1 California Academy of Sciences, San Francisco, CA 94118 USA California Academy of Sciences San Francisco United States of America

**Keywords:** Brazil, coral reefs, deep reefs, fish endemism, oceanic island, rebreather diving

## Abstract

During a recent expedition to St. Paul’s Rocks, Atlantic Ocean, a distinctive and previously unknown species of Anthiadinae was collected at a depth of 120 m. A genetic analysis indicated the undescribed species is a member of the genus *Tosanoides*, which was only known to occur in the Pacific Ocean. This new taxon is distinguishable from all other *Tosanoides* species by the following combination of characters: soft dorsal fin rays 15–16; anal fin rays 9; ventral scale rows 9–10; last dorsal spine the longest (instead first through fourth). Here *Tosanoidesaphrodite***sp. n.** is described and illustrated, only known from St. Paul’s Rocks.

## Introduction

The group commonly known as anthias fishes are classified within the serranid subfamily Anthiadinae Poey, 1861 (van der Laan et al. 2014), also historically known as “Anthiinae” (but see Carvalho-Filho et al. 2016; Pyle et al. 2016; Anderson et al. 2017; Eschmeyer and Fong 2018). In their review of the Atlantic and Eastern Pacific Anthiadine fishes, Anderson and Heemstra (2012) recognized 15 genera and 37 species, describing two new genera. Recently, one species of *Odontanthias* was described from St. Paul’s Rocks (Carvalho-Filho et al. 2016), an archipelago of small islets located around 940 km from Northeastern Brazil, in the Mid-Atlantic Ridge (Viana et al. 2009). St. Paul’s Rocks harbors one of the highest levels of endemism for reef fishes among Atlantic oceanic islands (Floeter et al. 2008, Pinheiro et al. 2018), even sheltering a genetic isolated population of a widespread species of Anthiadinae (Anderson et al. 2017).

The mesophotic coral ecosystems (MCEs; Hinderstein et al. 2010) of St. Paul Rocks were recently characterized by remote operated vehicles: Rosa et al. (2016) reported an assemblage of fishes at depths of 30–90 m depth dominated by two species, *Prognathodesobliquus* (Lubbock & Edwards, 1980) and *Chromisenchrysura* Jordan & Gilbert, 1882, while bryozoans, black corals and sponges were the main features of the benthic community. During a recent expedition to explore St. Paul Rocks that included diving to depths of up to 130 m, we collected specimens of a previously unknown Anthiadinae species. Here we describe it as a *Tosanoides* species, the first species of this genus to be recorded in the Atlantic Ocean, currently only known from St. Paul’s Rocks.

## Materials and methods

We collected using hand nets while diving on mixed-gas, closed-circuit rebreathers (Hollis Prism 2). We performed all counts using a microscope, with exception of vertebrae and caudal rays (primary, procurrent and rudimentary), which were counted from X-rays, and morphological characters were measured to the nearest 0.01 mm following Anderson and Heemstra (2012) and Pyle et al. (2016). We counted dorsal and ventral scale rows above and below lateral line to origins of dorsal and anal fins, respectively, including small truncate scales at bases of respective fins. Vertebral counts include the first vertebra fused to the skull, and the last vertebra fused to the hypural plate. Lateral-line scale counts include only those with pores. Caudal ray counts are presented as following: upper procurrent and rudimentary unbranched caudal rays + upper principal branched caudal rays + lower principal branched caudal rays + lower procurrent and rudimentary unbranched caudal rays. Rudimentary caudal rays are those only visible in the X-ray. In the description, counts and measurements for the holotype are presented followed by ranges for paratypes (in parentheses). Morphometric and meristic data for the type specimens are presented in Table 1. We deposited the specimens in the fish collection of the California Academy of Sciences (**CAS**), Universidade Federal do Espírito Santo (**CI-UFES**), Universidade Estadual de Campinas (**ZUEC**), Bernice Pauahi Bishop Museum (**BPBM**), U.S. National Museum of Natural History (**USNM**) and Museu de Zoologia da Universidae de São Paulo (**MZUSP**).

We sequenced and analyzed the Mitochondrial Cytochrome c oxidase subunit I (COI) DNA for the new species. DNA extraction and PCR amplification of the COI were performed following Weigt et al. (2012) protocols. We compared the DNA sequences to all species of Anthiadinae available in GenBank (*Tosanoidesobama*: KY370754; *Tosananiwae*: JF952878; *Odontanthiasperumali*: KR105805; *Plectranthiasjaponicus*: KP267602; *Sacuramargaritacea*: KF202522; *Anthiasanthias*: JQ774769; *Serranocirrhituslatus*: FJ584094; *Pseudanthiaspascalus*: FJ583931; *Pronotogrammusmartinicensis*: MF322587; *Baldwinellaaurorubens*: MG856775; *Acanthistiuspictus*: KY572857; *Meganthiasnatalensis*: KU176438; *Luzonichthysseaver*: KP110514; *Nemanthiascarberryi*: JQ350133; *Caprodonlongimanus*: DQ107894). GenBank accession number for the new species is MH817857.

## Results

### Tosanoides aphrodite sp. n.

Taxon classificationAnimaliaPerciformesSerranidae

http://zoobank.org/A2E4E1E2-0F05-4FAF-AC25-0CFE96ED9212

**Figure 1.**
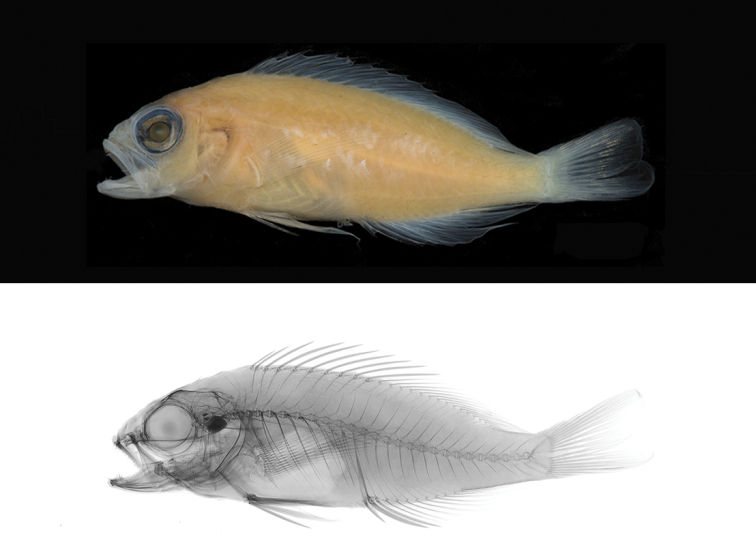
*Tosanoidesaphrodite* sp. n. holotype (CIUFES 3444), 56.8 mm SL, male, collected at a depth of 120 m in Saint Paul Rocks, Brazil. Photographs by J Fong.

**Figure 2.**
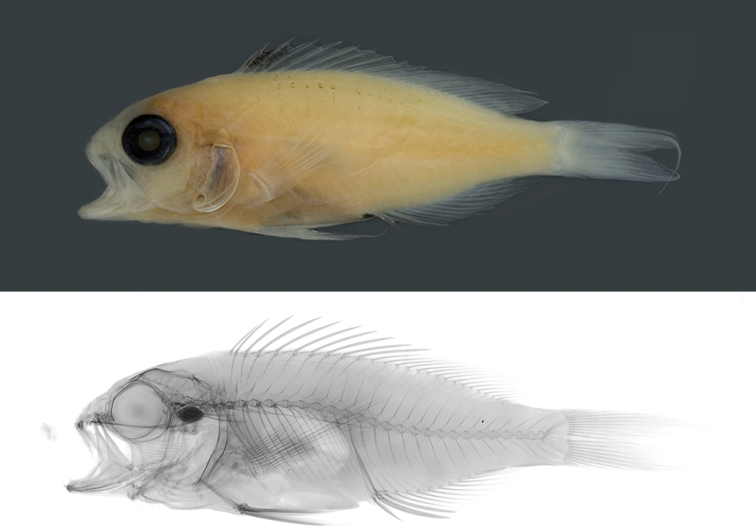
*Tosanoidesaphrodite* sp. n. paratype (CAS 244383), 47.5 mm SL, female, collected at a depth of 120 m in Saint Paul Rocks, Brazil. Photographs by J Fong.

**Figure 3.**
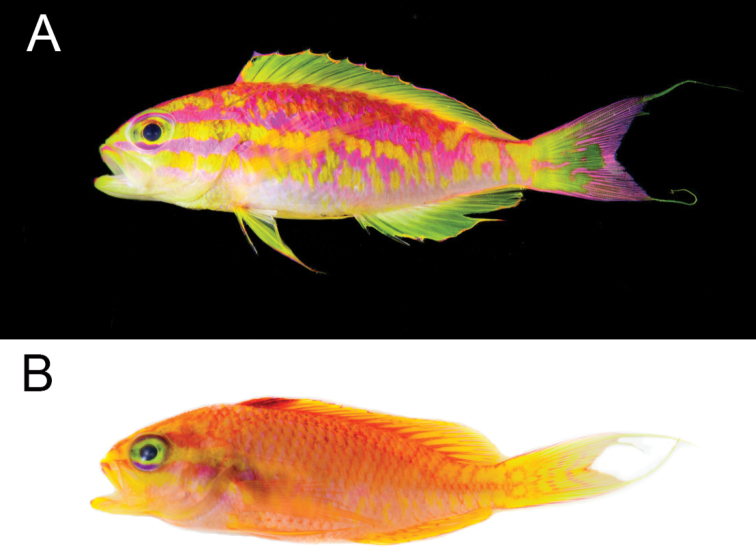
Fresh specimens of *Tosanoidesaphrodite* sp. n. collected in St. Paul’s Rocks, Brazil. **A** Male **B** Female. Photographs by LA Rocha.

**Figure 4.**
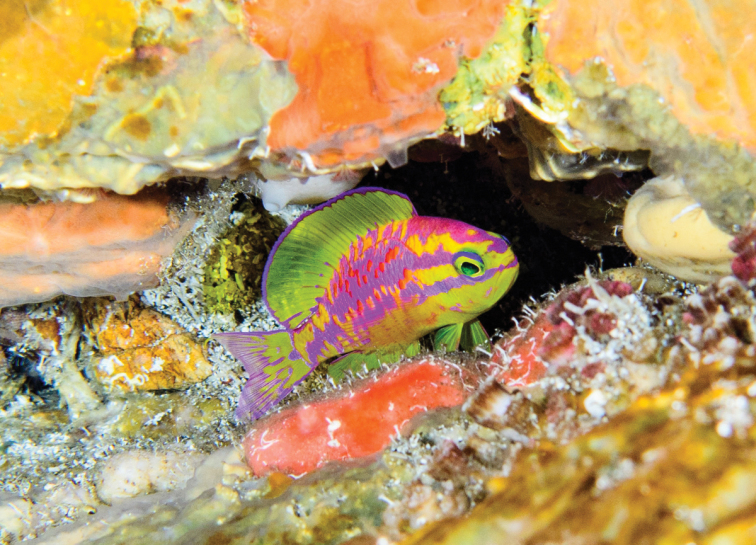
*Tosanoidesaphrodite* sp. n. in its natural environment, photographed at a depth of 120 m in St. Paul’s Rocks, Brazil. Photograph by LA Rocha.

Figures 1 , 2 , 3 , 4 , Table 1

#### Type locality.

Saint Paul’s Rocks, Brazil.

#### Material.

**Holotype.** CIUFES 3444 (Field number: CR 055). 56.8 mm SL, male, Saint Paul Rocks, Brazil. 00°56'N, 029°22'W, depth 120 m, collected by LA Rocha and HT Pinheiro using hand nets, 25 June 2017 (Figure 1). **Paratypes.**CAS 244382 (Field number: CR 071). 54.9 mm SL, male, Saint Paul Rocks, Brazil. 00°56'N, 029°22'W, depth 120 m, collected by LA Rocha and HT Pinheiro using hand nets, 25 June 2017. ZUEC 16842 (Field number: CR 077). 59.9 mm SL, male, Saint Paul Rocks, Brazil. 00°56'N, 029°22'W, depth 120 m, collected by LA Rocha and HT Pinheiro using hand nets, 25 June 2017. BPBM 41351 (Field number: CR 070). 57.1 mm SL, female, Saint Paul Rocks, Brazil. 00°56'N, 029°22'W, depth 120 m, collected by LA Rocha and HT Pinheiro using hand nets, 25 June 2017. MZUSP 123538 (Field number: CR 079). 38.9 mm SL, female (juvenile), Saint Paul Rocks, Brazil. 00°56'N, 029°22'W, depth 120 m, collected by LA Rocha and HT Pinheiro using hand nets, 25 June 2017. CAS 244383 (Field number: CR 078). 47.5 mm SL, female, Saint Paul Rocks, Brazil. 00°56'N, 029°22'W, depth 120 m, collected by LA Rocha and HT Pinheiro using hand nets, 25 June 2017 (Figure 2). USNM 440405 (Field number: CR 080). 33.1 mm SL, female (juvenile), Saint Paul Rocks, Brazil. 00°56'N, 029°22'W, depth 120 m, collected by LA Rocha and HT Pinheiro using hand nets, 25 June 2017.

#### Comparative material.

We compared *Tosanoidesaphrodite* to other Anthiadinae species using the keys for the Western Central Pacific Anthiadinae species in Heemstra and Randall (1999) and for the Atlantic and Eastern Pacific Anthiadinae species in Anderson and Heemstra (2012). Data from *Tosanoidesobama* Pyle, Green & Kosaki, 2016, *Tosanoidesflavofasciatus* Katayama & Masuda, 1980, and *Tosanoidesfilamentosus*Kamohara 1953 are from Pyle et al. (2016), Katayama and Masuda (1980), and Kamohara (1953), respectively.

#### Diagnosis.

The new species differs from all other Anthiadinae by the following combination of characters: Dorsal-fin spines X; last dorsal spine the longest, 1.8–2.2 in head length; dorsal-fin rays 15–16; 7^th^ dorsal ray the longest, 2.65–2.80 in head length; anal-fin rays 9; pored lateral-line scales 32–35; ventral scale rows 9–10; body slender and compressed, greatest depth 2.96–3.18 in SL, and the width 1.77–2.09 in depth. Our phylogenetic analysis shows the new species belongs to *Tosanoides*Kamohara 1953, from which it differs of the other known species by: a divergence of at least 12.35% at the cytochrome oxidase I gene, last dorsal spine the longest (instead first through fourth), fewer dorsal-fin rays (15–16 vs. 16–17), and more anal-fin rays (9 vs. 8 in the other *Tosanoides*).

#### Description.

Morphometric and meristic data for type specimens are provided in Table 1. Dorsal fin X, 15 (15–16), last soft ray branched to base; anal fin III,9, last soft ray branched to base; pectoral-fin rays 14 (14–15); pelvic-fin rays I,5; principal branched caudal rays 7 + 6 (7 + 6), upper procurrent and rudimentary unbranched caudal rays 9 (9), lower procurrent and rudimentary unbranched caudal rays 8 (8–9); pored lateral-line scales 34 (32–35); scale rows above lateral line to origin of dorsal fin 3; scale rows below lateral line to origin of anal fin 10 (9–10); gill rakers on upper limb 8, on lower limb 22; vertebrae 27 (10 precaudal + 17 caudal).

Body slender, compressed, its greatest depth 3.18 (2.96–3.13) in SL, the width just posterior to gill opening, 1.89 (1.77–2.09) in depth; head length 2.79 (2.88–3.52) in SL; snout short, its length 6.08 (4.88–7.12) in head; orbit diameter 3.34 (2.48–3.32) in head; interorbital convex, the least bony width 3.94 (2.78–4.03) in head; caudal peduncle depth 3.21 (2.33–3.56) in head. Mouth large and oblique; lower jaw not projecting beyond the upper when mouth closed; maxilla 2.15 (1.96–2.16) in head, diagonal (45°), and reaching the center of pupil. One pair of nostrils in front of each eye with no membranous tube or rims. One pair of pores on top of head between eyes, slightly anterior to center of eyes. Posterior margin of eye bordered with eight to ten pores. Lateral line very high, parallel with dorsal profile, forming an angle below last few dorsal rays and extending along middle of caudal peduncle to base of caudal fin.

Teeth in upper jaw villiform, forming a band broader anteriorly with two canines on each side, one externally directed forward and other internally directed backward, an outer row of approximately 14 slender canines on each side of jaw curved forward; lower jaw with a patch of villiform teeth anteriorly; two canines on each side anteriorly curved backwards and a third canine on each side facing forward and curved internally, an outer row of approximately 15 slender canines like those of upper jaw, pointing forward; small teeth on vomer and palatines; tongue pointed, smooth. Preopercle with a round angle, upper limb serrate with approximately 25 spinules, lower limb smooth; opercle with two flat spines, upper one longest and at apex; subopercle and interopercle smooth.

#### Color in life.

*Tosanoidesaphrodite* is sexually dichromatic. Males (Figure 3A): body pinkish and reddish, darker dorsally fading to white ventrally; two alternating bright yellow and pink stripes from anterior end of body through nape across the operculum, continuing to area below middle of spinous dorsal fin and becoming series of irregular spots on posterior third of body; third yellow stripe of similar pattern from lower jaw to caudal fin becoming series of irregular blotches under pectoral fin; eye yellow with bright pink upper and lower edges; snout and region anterior to eye bright yellow with a thin pink stripe extending dorsally to two thirds distance to origin of dorsal fin; scales on ventral portion of head and body with bright yellow margins; dorsal fin yellow with bright purplish pink margin; anal fin yellow also with bright pink margin from first to seventh ray and along posterior margin of last ray; pelvic fin yellow with pink anterior margin; caudal fin pink posteriorly and yellow with irregular pink markings anteriorly; filaments in upper and lower edges yellow; pectoral fin translucent yellow.

Females and juveniles (Figure 3B) predominantly reddish orange slightly darker dorsally; snout and region anterior to eye bright yellow with a thin red stripe extending dorsally to two thirds distance to origin of dorsal fin; two alternating yellow and red stripes from anterior end of body through nape to the operculum; third yellow stripe from lower jaw to base of pectoral fin; eye greenish yellow with bright purple upper and lower edges; scales on body with red margins; dorsal fins predominantly yellow with orange rays and dark red blotch covering first three dorsal spines; anal fin predominantly yellow with orange rays and orange margin; pelvic fin yellow with purplish red anterior margin; caudal fin yellow with pinkish orange margins and vertical lines forming ocellated and irregular markings; pectoral fin translucent orange.

#### Color in alcohol.

Straw-colored; fins transparent; eyes black (Figure 1). Females retain dark blotch on first three dorsal fin spines (Figure 2).

#### Etymology.

The name “*aphrodite*” refers to the ancient Greek goddess of love and beauty. While we were collecting the Aphrodite anthias, a large Six-gill shark (*Hexanchusgriseus*) came very close to both of us (HTP and LAR), but that didn’t divert our attention from the new exquisitely beautiful species, and we never even saw the shark (https://youtu.be/pSZrmoEwR0Q). The beauty of the Aphrodite anthias enchanted us during its discovery much like Aphrodite’s beauty enchanted ancient Greek gods.

#### Distribution and habitat.

*Tosanoidesaphrodite* is only known from Saint Paul’s Rocks, off Brazil. It was found on mesophotic coral ecosystems of the island, observed between 100 and 130 m depth while rebreather diving, and a single observation at 260 m depth, taken from a submersible dive. The species inhabits small crevices of complex rocky reefs (Figure 4). The ambient seawater temperature at the collecting depth (~ 120 m) varied between 13 and 15 °C during the two-week period we stayed in the area.

**Table 1.** Morphometric and meristic data for selected characters of type specimens of *Tosanoidesaphrodite* sp. n. Values of morphometric data are presented in mm.

Morphometrics	Holotype	Paratypes					
	CIUFES 3444	CAS 244382	ZUEC 16842	BPBM 41351	CAS 244383	MZUSP 123538	USNM 440405
Sex	Male	Male	Male	Female	Female	Female (juvenile)	Female (juvenile)
Total length (TL) in mm	78.3	74.1	86.9	73.4	63.9	52.5	46.2
Standard length (SL) in mm	56.77	54.89	59.88	57.09	47.55	38.91	33.09
Head length	20.32	18.58	20.79	19.54	16.53	12.76	9.39
Body depth	17.83	18.5	19.13	18.55	15.23	13.14	10.67
Body width	9.42	9.15	10.82	8.89	8.53	6.47	5.46
Snout length	3.34	2.98	4.26	3.04	2.88	1.87	1.32
Predorsal length	18.6	17.93	18.71	17.94	14.95	12.58	10.33
Preanal length	34.45	33.69	36.68	35.07	28.4	23.4	21.16
Base of dorsal fin	33.09	31.56	33.45	33.11	27.12	20.58	18.25
Base of anal fin	13.75	13.85	14.81	13.28	13.56	8.57	6.54
Orbit diameter	6.08	5.59	6.61	6.3	5.36	4.62	3.79
Interorbital width	5.16	5.39	5.16	5.09	4.49	3.66	3.38
Caudal peduncle depth	6.34	6.41	5.84	6.53	5.41	4.51	4.03
Pelvic spine	9.08	8.74	8.45	10.23	8.29	6.32	5.33
Pelvic fin	16.15	14.72	18.11	21.16	14.17	10.34	10.51
First dorsal spine length	4.16	3.72	5.07	4.37	4.78	2.29	2.46
Second dorsal spine length	5.33	4.59	5.97	4.92	5.96	3.41	2.98
Third dorsal spine length	7.21	6.21	8.1	6.28	6.92	4.25	4
Fourth dorsal Spine length	9.34	7.82	9.35	8.25	7.8	5.31	4.65
Fifth dorsal spine length	9.98	broken	10.89	8.55	7.92	5.62	4.85
Last dorsal spine length	10.39	10.19	11.16	9.12	7.95	5.87	5.03
Longest dorsal ray length (third)	10.98	9.25	12.63	11.04	8.32	6.34	5.33
First anal spine length	3.4	3.05	3.73	3.07	3.36	2.35	2.51
Second anal spine length	7.74	7.12	8.18	8.06	6.81	4.75	4.85
Third anal spine length	8.33	7.77	9.12	8.97	6.84	4.87	4.97
Longest anal ray length (third)	9.98	10.77	12.72	12.4	7.58	6.61	5.45
Caudal fin length	22.9	20.8	26.94	17.38	16.45	13.31	11.83
Pectoral fin length	20.86	19.55	20.72	20.62	16.82	12.77	11.34
maxilla	9.43	8.9	10.34	9.98	7.64	5.94	4.43
Meristics							
Dorsal spines	X	X	X	X	X	X	X
Dorsal rays	15	15	15	16	16	15	16
Anal spines	III	III	III	III	iii	III	iii
Anal rays	9	9	9	9	9	9	9
Pectoral rays	I 13 i	I 13	I 13 i	I 14	I 13 i	I 13	I 13i
Caudal rays	9+7+6+8	9+7+6+9	9+7+6+8	broken	9+7+6+8	9+7+6+9	9+7+6+8
Pored lateral line scales	24	23	23	25	loosen	26	23
Dorsal scale rows	3	3	3	3	3	3	3
Ventral scale rows	10	10	9	10	9	10	10
Gill rakers			8+22				

#### Comparative remarks.

The COI gene sequence of *Tosanoidesaphrodite* is 12.65% divergent from *Tosanoidesobama*, and on average 14–20% divergent from other genera of Anthiadinae. Because we do not have sufficient representation for members of this subfamily, no phylogenetic tree is provided here. In addition to the unique characters presented in the diagnosis section, *Tosanoidesaphrodite* also differs from *Tosanoidesfilamentosus* in having a shorter snout and larger orbit (4.9–7.1 and 2.5–3.3 vs 4.6 and 3.7 in HL, respectively). *Tosanoidesflavofasciatus* differs from *Tosanoidesaphrodite* in having a shorter fourth (2.55–2.75 vs. 2.02–2.40 in HL) and last (3.11–3.44 vs. 1.82 and 2.17 in HL) dorsal spine length, 7^th^ dorsal ray the longest instead the third, smaller third anal spine (2.65–2.80 vs. 1.89–2.62 in HL), and longer pectoral fin length (2.36–2.71 vs. 2.72–3.05 in SL). *Tosanoidesobama* also differs from *Tosanoidesaphrodite* in having a shorter fourth (2.4–2.68 vs. 2.02–2.40 in HL), fifth (2.4–2.88 vs. 1.94–2.29 in HL) and last (2.67–3.06 vs. 1.82–2.17 in HL) dorsal spine length, 7^th^ or 8^th^ dorsal ray the longest instead the third, and longer pectoral fin length (2.49–2.63 vs. 2.72–3.05 in SL).

## Discussion

Initially, differences in counts, body proportions, morphology of dorsal and caudal soft rays, and distribution (a single locality in the Atlantic versus wide range in the Pacific Ocean) made us believe that *T.aphrodite* constituted a new genus. However, preliminary genetic analysis based on the COI gene placed *T.aphrodite* between two pairs of *Tosanoides* species (including an undescribed new species from Pohnpei, Micronesia; Pyle et al., submitted). Therefore, we provisionally place this new species in *Tosanoides*, but future genetic analyses with multiple loci might clarify the Anthiadinae classification and change the generic placement of *T.aphrodite*.

The isolation of Saint Paul’s Rocks, from both the American and African coastlines, and its extremely small size, are likely the main causes for the high endemism and low diversity in the local marine biodiversity. Previously, there were seven endemic reef fishes that were exclusively found at St. Paul’s Rocks, while another six restricted range species were shared with the Brazilian oceanic islands of Fernando de Noronha and Rocas Atoll (Pinheiro et al. 2018). Thus, *Tosanoidesaphrodite* is the eighth endemic to St. Paul’s Rocks, adding to the uniqueness of this locality. However, like other inhabitants of the poorly sampled mesophotic reefs, the Aphrodite Anthias might have a wider distribution. For example, another Anthiadinae, *Anthiasasperilinguis* Günther 1859, is widespread along deep reefs of the western Atlantic (usually below the MCEs), and is also found at St. Paul’s Rocks, where it shows a slight genetic divergence (Anderson et al. 2017). The few studies of deep reefs in the southwestern Atlantic are mostly restrict to shallower mesophotic coral ecosystems between 30 and 80m depth (Pinheiro et al. 2015, Rosa et al. 2016, Simon et al. 2016), what limits the understanding of the biogeography of deep fishes in the province.

## Supplementary Material

XML Treatment for Tosanoides aphrodite
